# Analgesic effect of cathodal transcranial current stimulation over right dorsolateral prefrontal cortex in subjects with muscular temporomandibular disorders: study protocol for a randomized controlled trial

**DOI:** 10.1186/s13063-015-0938-0

**Published:** 2015-09-17

**Authors:** Rivail Almeida Brandão Filho, Abrahão Fontes Baptista, Renata de Assis Fonseca Santos Brandão, Francisco Monteiro Meneses, Jeffrey Okeson, Eduardo Pondé de Sena

**Affiliations:** Postgraduate Program of Interactive Processes of Organs and Systems, Health and Science Institute of Federal University of Bahia and University of State of Bahia, Avenida Reitor Miguel Calmon, S/N, Vale do Canela, Salvador, Bahia CEP 40110-902 Brasil; University of the State of Bahia, Rua Silveira Martins, 2555, Cabula, Salvador, Bahia CEP: 41.150-000 Brasil; Functional Electrostimulation Laboratory, Department of Biomorphology, Health and Science Institute, Federal University of Bahia, Avenida Reitor Miguel Calmon, S/N, Vale do Canela, Salvador, Bahia CEP 40110-902 Brasil; Department of Oral Health Science, University of Kentucky College of Dentistry, Lexington, KY USA; Department of Bioregulation, Health Sciences Institute, Federal University of Bahia, Avenida Reitor Miguel Calmon, S/N, Vale do Canela, Salvador, Bahia CEP 40110-902 Brasil

**Keywords:** Anxiety, Chronic pain, Temporomandibular, Musculoskeletal diseases, Electrical stimulation, Brain prefrontal cortex

## Abstract

**Background:**

Temporomandibular disorders are a group of orofacial pain conditions that are commonly identified in the general population. Like many other chronic pain conditions, they can be associated with anxiety/depression, which can be related to changes in the activity of the dorsolateral prefrontal cortex. Some studies have demonstrated clinical improvement in subjects with chronic pain who are given therapeutic neuromodulation. Transcranial direct current stimulation is a noninvasive brain stimulation technique that allows the modulation of neuronal membranes. This therapy can enhance or inhibit action potential generation in cortical neurons. In some instances, medications acting in the central nervous system may be helpful despite their adverse side effects. It is important to determine if cathodal transcranial direct current stimulation over the dorsolateral prefrontal cortex, an area that modulates emotion and motor cortex excitability, has an analgesic effect on chronic temporomandibular disorders pain.

**Method/design:**

The investigators will run a randomized, controlled crossover double blind study with 15 chronic muscular temporomandibular disorder subjects. Each subject will undergo active (1 mA and 2 mA) and sham transcranial direct current stimulation. Inclusion criteria will be determined by the Research Diagnostic Criteria for Temporomandibular Disorders questionnaire, with subjects who have a pain visual analogic scale score of greater than 4/10 and whose pain has been present for the previous 6 months, and with a State-Trait Anxiety Inventory score of more than 42. The influence of transcranial direct current stimulation will be assessed through a visual analogic scale, quantitative sensory testing, quantitative electroencephalogram, and the State-Trait Anxiety Inventory score.

**Discussion:**

Some studies have demonstrated a strong association between anxiety/depression and chronic pain, where one may be the cause of the other. This is especially true in chronic temporomandibular disorders, and breaking this cycle may have an effect over the symptoms and associated dysfunction. We believe that by inhibiting activity of the dorsolateral prefrontal cortex though cathodal transcranial direct current stimulation, there may be a change in both anxiety/depression and pain level. Transcranial direct current stimulation may emerge as a new tool to be considered for managing these patients. We envision that the information obtained from this study will provide a better understanding of the management of chronic temporomandibular disorders.

**Trial registration:**

This trial was registered at clinicaltrials.gov on 24 May 2014 (Identifier: NCT02152267).

## Background

Pain is among the most common complaints reported by patients seeking care in a hospital setting or in a primary care unit. Patients with pain due to temporomandibular disorders (TMD), which are musculoskeletal disorders of the masticatory structures, are very common in the general population. Temporomandibular and craniofacial disorders are so prevalent that the International Headache Society has developed a classification for the many different types of head pain. The third edition of the International Classification of Headache Disorders was published in 2013 [1].

There are several strategies used to manage patients with TMD. Since there are many types of TMD, it is critical that clinicians make the proper diagnosis so that appropriate treatment is selected. Treatment strategies range from patient education and self-management to dental procedures and surgery. The most common symptom associated with TMD is muscle pain, which is often managed by cognitive awareness, behavioral changes, and stress management [2].

A study using functional brain imaging showed that, although the majority of temporomandibular disorders are associated with muscle pain, as they become chronic they are likely to have a centralnervous system (CNS) component [3]. Supporting this concept are behavioral studies that have demonstrated that TMD is often associated with psychopathology [4, 5].

It is now commonly accepted that chronic pain involves significant modifications in central neuronal excitability. In a fibromyalgia study using transcranial magnetic stimulation (TMS), the authors found higher thresholds in both sides of the resting motor cortex with lower up regulation responses. Furthermore, they found lower intracortical facilitation and inhibition. The effects of neural modulation to manage chronic pain are presently being studied [6].

Transcranial direct current stimulation (tDCS) modulates neuronal membranes of cortical neurons, enhancing or inhibiting their action potentials. Experimental animal studies have shown that anodal stimulation depolarizes the nerve membrane, which results in a long-term potentiation in the stimulated area [7, 8]. Some studies have shown pain reduction in subjects with fibromyalgia and chronic pain using neuromodulation [9–13]. Some suggest that anodal stimulation over the motor cortex decreases pain intensity by modulating the activity in the neuronal networks responsible for pain. Another study that evaluated the role of the thalamic ventral caudal nucleus in nociception, suggested a decrease of thalamic pain control output capacity in chronic pain patients [14]. Transcranial direct current stimulation may facilitate the descending inhibition of pain; however, the current evidence is weak [15].

Others studied the effects when transcranial direct current stimulation (tDCS) is placed over the dorsolateral prefrontal cortex (DLPFC) [16]. This area is involved with anxiety and depression without direct analgesic effect. Although not the objective, the authors found anxiety modulation after tDCS application over the DLPFC, when the cathode was placed over the right side (F4) and the anode on the left side (F3). These results show weak evidence of tDCS effects on anxiety, but they do suggest the need for additional investigation [16]. The modulation of DLPFC may also have analgesic effects through its excitatory interaction with the primary motor cortex [17].

If cathodaltDCS over the DLPFC has an analgesic effect through modulation of negative emotions, another important issue is to determine the best stimulation intensity to achieve this goal. Previous tDCS studies have used 1 or 2 mA, with 2 mA being more common. There is discussion regarding which intensity is more appropriate with cathodal tDCS. Some authors found that either 2 mA cathodal or anodal stimulation led to increased excitability of the primary motor cortex, whereas 1 mA stimulation had a specific effect, with cathodal current decreasing cortical excitability [18].

An advantage of tDCS is that it has minimal side effects. The most common adverse effects are itching, tingling, and mild headache that resolve just after the stimuli is terminated. These adverse effects depend on the intensity and duration of the treatment session. The most common protocols use a lower intensity (1 or 2 mA) with a treatment session duration of 20 or 30 minutes. To access tolerability and safety of tDCS, a side effect questionnaire has been developed [19].

## Aims

The main objective of this study is to investigate whether cathodal tDCS over the right DLPFC has analgesic effects in subjects with muscular TMD pain. This study will: (1) evaluate the tDCS effect on pain perception and pressure threshold; (2) evaluate whether tDCS has any effect on modifying anxiety and stress; (3) evaluate whether an EEG’s waves change from pre- to post-tDCS; (4) investigate whether there is an association of pain intensity with anxiety and EEG data pre- and post-tDCS; (5) compare the effects of 1 mA and 2 mA over pain intensity, anxiety, stress, and EEG.

## Methods

### Study design

This is a randomized, controlled crossover double blind study with one group undergoing three different interventions. Treatment order will be determined randomly. The CONSORT guidelines from 2010 to clinical trials will be used to guide the procedures [20].

### Eligibility criteria

To be included in this study, subjects must: (1) provide informed consent to participate in the study; (2) be between 18–60 years old, regardless of gender; (3) have a diagnosis of muscular TMD pain according to IA and IB, Axis I of Research Diagnostic Criteria for Temporomandibular Disorders (RDC/TMD); (4) have a visual analog scale (VAS) pain score of 4 or greater, present regularly for 6 months or longer; (5) have a State-Trait Anxiety Inventory (STAI) score of more than 42.

Subjects will be excluded from this study if they (1) are pregnant; (2) have contraindications to tDCS, such as metal implants on the head or implanted brain devices; (3) have a history of alcohol or drug abuse within the past 6 months as self-reported; (4) have used carbamazepine within the past 6 months as self-reported; (5) have a history of epilepsy, stroke, moderate-to-severe traumatic brain injury, or severe migraines; (6) have a history of neurosurgery as self-reported; (7) have a history of temporomandibular joint problems such as disc displacement, arthralgia, osteoarthritis, or osteoarthrosis (Axes I, II, and III diagnosis from RDC/TMD); (8) have a major psychiatric disorder such as schizophrenia or bipolar disorder; (9) have had any other previously diagnosed disorder that has pain symptoms similar to muscle TMD, such as fibromyalgia.

### Intervention groups

Each subject will be tested to determine the effect of cathodal tDCS over the DLPFC on anxiety using two different currents, 1 mA and 2 mA.

### Intervention A

This intervention will involve 1 mA tDCS, cathodal over the right DLPFC (F4). The anode will be placed over the contralateral supraorbital area (Fp1). Stimulation will be applied over a 20-minute period.

### Intervention B

This intervention will use the same parameters as intervention A, but using 2 mA.

### Intervention C

This will be the sham intervention. Stimulation electrodes will be placed at the same areas as for interventions A and B, but the current will be applied only for the first 30 seconds, after which it will be reduced gradually until it reaches zero. This method has been shown to be effective in previous studies [21].

In all the intervention groups, tDCS will be applied using 35 cm^2^ (5×7 cm) sponge electrodes soaked in saline solution (140mMolNaCl, water dissolved in Mille-Q). Anodal and cathodal electrodes will be connected in the tDCSdevice (Soterix Medical 1 × 1 tDCS 1300A, USA). Two 9-V batteries will deliver current.

All subjects will receive only one stimulation session for each type of intervention (IA, IB and IC), for a total of three sessions. After each tDCS session, the subject will complete a questionnaire to record any adverse affects. A trained researcher (RABF) will perform each procedure. One researcher who is responsible for the general supervision (EPS) during testing will hold the randomization list, and only the clinician responsible for the tDCS stimulation will know which intervention group is being applied. All clinical and neurological assessments will be performed at the Functional Electrostimulation Laboratory at Federal University of Bahia (Salvador, Brazil).

As this is a crossover study with only four weekly sessions, we believe that there will not be a compliance problem. Furthermore, since side effects are minimal, there is a low risk of dropouts due to this reason.

### Control group

As a crossover study, each subject will be his/her own control. This choice is supported by studies that evaluated motor cortical excitability with transcranial magnetic stimulation (TMS) and found that a single tDCS session is able to modify cortical excitability for up to 1.5 hours after the end of stimulation. This suggests that a carryover effect is not an issue [22, 23]. Furthermore, EEG data is one of our outcome measures, and individual characteristics are crucial in this analysis [24], being minimized by a crossover design.

### Outcomes

The primary outcome of this study will be the extent to which this method will change baseline pain intensity (VAS). The subjects will record their experience on a daily basis in a pain diary. A weekly response average will be calculated before the first intervention and after each session. The averages will be used to compare the VAS values before and after each intervention. A decrease of 50 % will be considered a satisfactory clinical improvement.

Secondary outcomes will be the degree of change from baseline in power density of alpha and theta EEG bands. Other outcome measures will be pain pressure threshold (PPT), mechanical pain thresholds (MPTs), and STAI. Increase in PPT and mechanical thresholds indicate an improvement in subject pain condition; a decrease in STAI indicates a better emotional state.

### Participant timeline

**Fig. 1:**
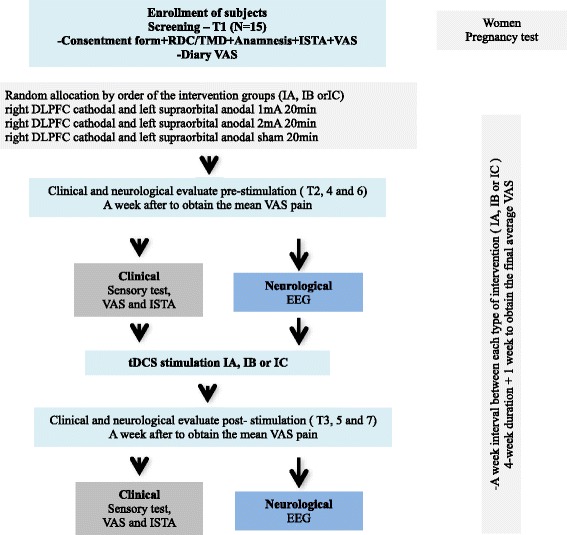
Flow of study procedures

All study procedures are depicted in Fig. 1, and the visit summary is given in Table 1.

**Table 1:** Visit summary

	V1	V2	V3	V4
	Baseline/consent	1^st^ session	2^nd^ session	3^rd^ session
Consent form	X			
RDC/TMD	X			
VAS pain diary	X	X	X	X
VAS pain (pre-tDCS)		X	X	X
Sensory testing (pre-tDCS)		X	X	X
EEG recording (pre-tDCS)		X	X	X
tDCS stimulation (IA, IB, or IC)		X	X	X
Adverse effects questionnaire		X	X	X
VAS pain (post-tDCS)		X	X	X
ISTA	X	X	X	X
EEG recording (post-tDCS)		X	X	X
Sensory testing (post-tDCS)		X	X	X
*Approx. time*	*1 hour*	*3 hours*	*3 hours*	*3 hours*

### Sample size

Following IMMPACT (Initiative on Methods, Measurement, and Pain Assessment in Clinical Trial) [25] recommendations for clinical trials involving chronic pain, we considered our primary outcome measure to be pain intensity changes, assessed through a VAS. We achieved a sample size of 15 subjects (G*Power 3.1), considering a reduction of 50 % (effect size of 0.5), a study power of 80 %, an alpha value of 5 % (*P* < 0.05), and six measures (three pre- and post-measures) on three intervention groups (placebo, and tDCS 1 mA and 2 mA).

### Recruitment

The subjects will be identified from the database of a public dental reference center (COAT – Faculty of Dentistry, Federal University of Bahia, Brazil).

### Allocation of interventions

Subjects will be consecutively randomized in order of the interventions using the tool from randomization.com (DallalGE, http://www.randomization.com). This study will use the second generation suggested for crossover studies. Only the investigators assigned to apply tDCS will have access to the randomized list. This list will be opened only when all data are available and analyzed, unless a subject has a side effect that justifies the interruption of the procedure.

### Data collection procedure

After COAT institutional authorization, subjects will be contacted by phone to ascertain their interest. All interested participants will complete a pre-screening questionnaire. Each subject will be scheduled for an appointment in order to further determine if they meet the inclusion criteria. If they do, they will sign an informed consent and be formerly enrolled in the study.

Women of childbearing age will be required to take a urine pregnancy test during the screening process. If a subject becomes pregnant during the course of the study, she will be withdrawn. During this initial appointment, the subject will be asked to complete a daily pain VAS diary for 7 days. The average VAS score from this data will be used as a baseline pain value.

During a second visit (T2) all clinical and neurological assessments will be performed before and after the tDCS stimulation. The clinical assessments will be measured by VAS pain scores, anxiety measures, and quantitative sensory tests. Neurophysiological assessment will be measured by using an EEG to analyze cortical activity. Each patient will be clinically and neurologically assessed before and after the treatment session resulting in six data collection points (T2 to T7).

All subjects will be treated during a single session for three different interventions (IA, IB,or IC) with a washout period of 7 days between treatments in order to avoid any residual effects. As reported earlier, a single tDCS session usually is able to modify cortical excitability for 1 hour after stimulation [22, 23].

### Procedure details

Recently, a new version of RDC/TMD, now called Diagnostic Criteria for Temporomandibular Disorders (DC/TMD), was published [26]. However, since the new DC/TMD was not translated and validated into Brazilian Portuguese, we have decided to use the already-validated Brazilian version from 2004 [27]. This instrument will only be used to determine inclusion criteria.

RDC/TMD evaluation has two axes, one for assessing clinical characteristics (Axis I), and the other for assessing the psychosocial aspects (Axis II). As the specific instrument used to assess anxiety and depression will be the STAI scale, only Axis I of RDC/TMD will be used. The RDC/TMD has three diagnostic groups: (1) muscle disorders; (2) disc displacements; (3) arthralgia/arthritis/arthrosis [28]. Subjects who present only with muscle disorders will be included.

#### Visual analog scale (VAS) for pain

The VAS allows us to convert subjective sensations, such as pain, into numerical data. A 10-cm scale, where 0 cm is no pain and 10 cm is the worst imaginable pain, will be used, and the subjects will be asked to mark a point on the scale representing their pain intensity. This instrument will be completed everyday in the 7-day period after their acceptance. An average pain intensity score will be calculated as a baseline for the first treatment intervention. Averages for each week will be used to analyze if there will be any treatment carryover effect. The VAS scores will be recorded before and after each treatment session to verify treatment effect.

#### State-Trait Anxiety Inventory (STAI)

The STAI is used to evaluate objectively both aspects of anxiety: trait and state. Analyzing trait will show the personality, auto image, and the way that the subject realizes and perceives threatening situations. State anxiety is a transitory emotional state, in response to environmental stimuli such as tachycardia, sweating, nausea, and cramps. The questionnaire addresses 20 possible sensations for each. We will analyze values from 1 to 4, where 1 is never, 2 sometimes, 3 often, and 4 always. The results reveal whether the subject tends to present low levels of anxiety (20–40 points), middle levels of anxiety (41–60 points), or high levels of anxiety (61–80 points).

#### Quantitative sensory testing

Mechanical perception and pain threshold will be tested using Semmes-Weinstein monofilaments (0.008 to 300 g/mm^2^). Monofilament application will be at a masseter tender point with the control being the contralateral thenar area (the palm of the hand at the base of the thumb). Hairs will be applied gradually, until the subject perceives the stimulus (sensory perception threshold). The intensity will be increased until the subject describes it as painful (pain threshold). The thresholds will be taken at the lowest force that causes sensory/pain perception. The side of the most painful masseter will be used for both assessments.

#### Pain pressure threshold (PPT)

PPT will be determined by applying blunt pressure by a hard-rubber probe, using an approved device (EMG 1630WF, EMG System, Brazil). During the test, pressure will be applied to the same area as in the mechanical perception and pain threshold tests, with increasing intensity at a rate of 1 kg/second. When the subject reports that he/she feels any pain, the procedure will be stopped. A computer program (EMGLab, EMG System, Brazil) will automatically record the value. This procedure will be repeated three times to achieve an average.

These tests will take approximately 7–10 minutes to be completed.

#### Electroencephalogram (EEG)

The EEG is a powerful tool to assess changes related to anxiety [29]. It will measure the brain’s electrical current intensity, using the analysis of alpha and theta waves. Alpha waves are related to physical relaxation and are higher when the subject keeps his/her eyes closed. Alpha power density is reduced when the eyes are open. The same can be seen in relaxation and alertness respectively. Theta waves are related to awareness states and are higher chronic pain conditions.

For this study, a 32-channel EEG (BrainNet BNT 36, EMSA, Brazil) will be used. Data will be band pass filtered (0.5 to 50 Hz) and acquired at 200 Hz. The channel location will be used according to the International 10–20 EEG System. Wave power and frequency will be checked before and after tDCS stimulation. The reference electrodes will be located at Cz. The data will be analyzed in MATLAB (The Mathworks, Inc., Natick, MA, USA). After signal filtering, data will be epoched into 1.28-second segments to standardize artifact removal. Artifacts will be removed using EEGLab (the semi-automated rejection EEGLab). Epochs containing extreme values (above −750 and below 750 μV) will be rejected using independent component analysis (ICA), removing the first component. After artifact removal, a frequency analysis will be run based on the following band widths: a) theta (4–8 Hz), b) alpha (8–12 Hz), and c) beta (13–30Hz).

### Safety monitoring

At each stimulation session, subjects will complete a questionnaire to evaluate potential adverse effects of tDCS (tingling, burning sensation, headache, neck pain, mood alterations) on a 5-point scale. Subjects will be asked whether they experienced any side effects in an open-ended manner, and they will be specifically asked about headache, neck pain, scalp pain, burning sensations, tingling, skin redness, sleepiness, trouble concentrating, and acute mood change.

### Study variables

The dependent variables will be (1) VAS for pain intensity, (2) STAI, (3) quantitative sensory testing, and (4) EEG quantitative analysis. The independent variables will be (1) cathodal tDCS over DLPFC (1 mA and 2 mA) and (2) sham treatment.

### Statistical methods

The data will be recorded and analyzed by a blinded biostatistician. First, a descriptive analysis of variables will be performed. Data distribution will be analyzed using the Shapiro-Wilk test. To check carryover effects between sessions, we will carry out a *t*-test or a Wilcoxon rank sum, depending on the normal distribution.

Outcome measures will be compared both between and within groups considering pre- and post-interventions with repeated measures analysis of variance or nonparametric correspondence tests (Friedmann and Mann–Whitney tests). Clinical data will be correlated to EEG data through correlation tests (Spearman or Pearson). Significance will be established at an alpha value of 5 %, with a study power of 80 %. All data will be analyzed with intention-to-treat.

### Ethical aspects

This protocol will be performed in accordance with resolution 466/2012 of the Brazilian National Research Ethics Committee (CONEP – ConselhoNacional de Éticaem Pesquisa). The Ethics Committee of Maternidade Climério de Oliveira, Federal University of Bahia approved this protocol (process number: 659.0460). Ten sessions of the best treatment protocol found in this study will be offered to all the participants at the end of the analysis.

### Technical and scientific resources

The two important goals of this research are to establish the most effective protocol for tDCS and to demonstrate a therapy that may be useful for TMD patients. The use of tDCS for TMD is feasible in clinical practices that have many patients presenting with this disorder. tDCS is noninvasive, low cost, and easy and rapid to implement. Further, this treatment has minimal adverse effects.

## Discussion

Most strategies for treating TMD are aimed directly at the craniofacial muscles, applying physiotherapy on the temporomandibular joint (TMJ) and/or on the jaws and on the occlusion of teeth [2]. Since chronic TMD is likely to have a central nervous system component in its etiology, some drugs, such as tricyclic antidepressants, may have a positive effect on TMD symptoms. However, after long-term use, some patients become resistant to the effects of these medications, resulting in the return of pain. In addition, some of these medications have undesirable side effects [30].

Based on past studies that have demonstrated changes in brain activity in chronic pain patients [6, 11], it is promising that this treatment may be helpful in chronic TMD. If this is true, tDCS may emerge as an adjunctive therapeutic tool that can be coupled with other effective treatments already in use.

There seems to be a correlation between anxiety and muscular hyperactivity [5], yet there is no evidence that tDCS placed over the emotional areas of the brain decreases anxiety [16]. This study may help shed light on whether tDCS over the DLPFC has an analgesic effect when stimulating the emotional areas and if different amplitudes result in different effects. Perhaps neuromodulation by tDCS over the DLPFC may decrease anxiety and consequently muscular hyperactivity. This may also lead to a reduction of chronic TMD pain.

The tDCS technique has shown promising results for the treatment of chronic pain associated with several types of disorders [9, 13, 31]. We are hopeful that tDCS may be effective for the management of painful TMD.

## Trial status

We are recruiting subjects.

## Abbreviations

CNS: central nervous system
DLPFC: dorsolateral prefrontal cortex
EEG: electroencephalogram
RDC/TMD: Research Diagnostic Criteria for Temporomandibular Disorders
PPT: pain pressure threshold
STAI: State-Trait Anxiety Inventory
tDCS: transcranial direct current stimulation
TMD: temporomandibular disorders
TMJ: temporomandibular joint
VAS: visual analog scale
